# Canola Cake as a Potential Substrate for Proteolytic Enzymes Production by a Selected Strain of *Aspergillus oryzae*: Selection of Process Conditions and Product Characterization

**DOI:** 10.1155/2013/369082

**Published:** 2013-12-25

**Authors:** Adriana C. Freitas, Ruann J. S. Castro, Maria A. Fontenele, Antonio S. Egito, Cristiane S. Farinas, Gustavo A. S. Pinto

**Affiliations:** ^1^Department of Food Engineering, Federal University of Maranhão, Rua Urbano Santos S/N, 65900-410 Imperatriz, MA, Brazil; ^2^Department of Chemical Engineering, Federal University of Ceará, Avenida da Universidade 2853, 60020-181 Fortaleza, CE, Brazil; ^3^Embrapa Tropical Agroindustry, Rua Doutora Sara Mesquita 2270, 60511-110 Fortaleza, CE, Brazil; ^4^Embrapa Goats and Sheep, Estrada Sobral/Groaíras km 04, 62010-970 Sobral, CE, Brazil; ^5^Embrapa Instrumentation, Rua XV de Novembro 1452, 13560-970 São Carlos, SP, Brazil

## Abstract

Oil cakes have excellent nutritional value and offer considerable potential for use in biotechnological processes that employ solid-state fermentation (SSF) for the production of high value products. This work evaluates the feasibility of using canola cake as a substrate for protease production by a selected strain of *Aspergillus oryzae* cultivated under SSF. The influences of the following process parameters were considered: initial substrate moisture content, incubation temperature, inoculum size, and pH of the buffer used for protease extraction and activity analysis. Maximum protease activity was obtained after cultivating *Aspergillus oryzae* CCBP 001 at 20°C, using an inoculum size of 10^7^ spores/g in canola cake medium moistened with 40 mL of water to 100 g of cake. Cultivation and extraction under selected conditions increased protease activity 5.8-fold, compared to the initial conditions. Zymogram analysis of the enzymatic extract showed that the protease molecular weights varied between 31 and 200 kDa. The concentrated protease extract induced clotting of casein in 5 min. The results demonstrate the potential application of canola cake for protease production under SSF and contribute to the technological advances needed to increase the efficiency of processes designed to add value to agroindustrial wastes.

## 1. Introduction

Proteases are one of the three largest groups of industrial enzymes and have a wide range of applications in the food, textile, and pharmaceutical industries [1]. Sources of proteases include plants, animals, and microorganisms. However, proteases from plant and animal sources are unable to meet global demand, as a result of which there is a need to develop efficient processes for microbial protease production. In fact, of the hundreds of enzymes currently used industrially, over half are derived from fungi and more than one-third from bacteria, with the remainder originating from animal (8%) and plant (4%) sources [2]. Microbial proteases account for approximately 40% of total worldwide sales of enzymes [3].

Processes that can be used for microbial protease production include submerged fermentation (SmF) and solid-state fermentation (SSF). The latter is particularly advantageous for enzyme production by filamentous fungi, since it simulates the natural habitat of these microorganisms [4, 5]. Another advantage of SSF is that agroindustrial wastes (oil cakes, bagasse, wheat bran, etc.) can be used as the solid substrate, acting as sources of both carbon and energy. However, certain operational limitations of SSF, such as difficulty in controlling the moisture level of the substrate and avoiding heat buildup, have limited its industrial application. Characterization of each individual microorganism, in terms of the influence of temperature and substrate moisture content on the kinetics of growth and product formation, is essential for SSF process scale-up [6]. The strains that have been considered for the production of proteases under SSF include *Aspergillus *and* Penicillium* species, amongst others [7–10].

Oil cakes have high nutritional value and are potentially valuable substrates for use in biotechnological SSF processes for the manufacture of high value products [11, 12]. The development of new canola varieties has enabled edible oils to be obtained from rapeseed, which is now the world's third largest source of vegetable oil. The by-product of oil extraction, canola cake, is not only a rich source of nitrogen, carbon, and minerals but also abundant and inexpensive [12]. However, despite the potential of *Aspergillus *strains and their recognized applications in industrial enzyme production, to the best of our knowledge there have been no studies concerning the characterization of these fungi for the production of proteolytic enzymes using canola cake as solid substrate in SSF.

The aim of the present study was to evaluate the suitability of canola cake as a substrate for protease production using SSF. Following initial screening of different *Aspergillus* strains, an *A. oryzae* strain was selected for thorough characterization in terms of protease production under different process conditions. The effectiveness of the proteolytic enzymatic extract was determined by its ability to clot milk.

## 2. Materials and Methods

### 2.1. Raw Material

Canola cake, a residue remaining after extraction of the oil, was kindly provided by Celena Alimentos (Eldorado do Sul, Rio Grande do Sul, Brazil). The cake was used as SSF substrate without any pretreatment.

### 2.2. Microorganisms

The following strains from the collection maintained by Embrapa Tropical Agroindustry (Fortaleza, Brazil) were evaluated for protease production: *Aspergillus niger* CCBP 002, *A. niger* CCT 0916, *A. niger* IOC 4220, *A. niger* IOC 3883, *A. niger* IOC 4222, *A. niger* IOC 207, and *A. oryzae *CCBP 001. The strains were kept on dry sand at −18°C and were activated in basic agar slants, as described by [13]. Conidia from the second activation step were suspended in Tween 80 solution (0.3%, v/v) that had been sterilized at 121°C for 15 min.

### 2.3. Preparation of Inoculum

A 1 mL volume of the spore suspension was inoculated into a 250 mL Erlenmeyer flask containing ground corn cob and a nutrient medium [13] and incubated for 5 days at 30°C. The spores were then dispersed with 40 mL of 0.3% (w/v) Tween 80, using a sterile stick under aseptic conditions. The concentration of spores in the suspension was estimated by counting in a Neubauer chamber.

### 2.4. Solid-State Fermentation

SSF cultivations were performed using 40 g of canola cake medium in 500 mL Erlenmeyer flasks that had been sterilized at 121°C for 15 min and then inoculated with the spore suspension. The contents were homogenized and the flasks were incubated for 96 h, with samples collected every 24 h. Extraction of the crude enzymatic extracts was accomplished by the addition of 100 mL of 0.2 mol/L acetate buffer to the fermented media, followed by incubation at 30°C for 1 h. The crude extracts were filtered, and the supernatants were used for further analysis.

### 2.5. Screening for Fungal Strain Selection

In this initial fermentation, 75 mL of water was added to each 100 g of canola cake, and the inoculum size was 10^7^ spores/g. Samples were withdrawn after every 24 h interval during a period of 96 h incubation at 30°C. The criterion for strain selection was based on protease activity.

### 2.6. Effects of Process Parameters on Protease Production

The strategy adopted for the selection of SSF conditions was to evaluate each variable individually and then select the best value for incorporation in the next variable selection step. The parameters and values employed were as follows: initial water volume added to the solid medium (10, 20, 30, 40, 50, 75, 100, 125, and 150 mL), inoculum size (10^4^ to 10^7^ spores/g of medium), and incubation temperature (15, 20, 30, 35, and 40°C).

### 2.7. Influence of Extraction pH and Determination of the Optimum pH for the Enzyme

A set of experiments were carried out in order to identify the best pH for recovery of protease. The buffers used were 0.2 mol/L acetate buffer (for pH 4.0 and 5.0) and 0.05 mol/L sodium phosphate buffer (for pH 6.0, 7.0, and 8.0). The optimum pH for protease was determined by assaying the activity at different pH values (from 4.0 to 8.0, adjusted using the same buffers).

### 2.8. Preparation of the Protease Extract for Characterization Studies

The protease enzymatic extract obtained under the selected conditions was concentrated using a precipitation step. Ammonium sulfate was added to the crude enzymatic extract in amounts corresponding to 40, 60, 80, and 100% saturation. The salt was added slowly, with gentle stirring, and the mixture was maintained at 4°C for 2 h for enzyme precipitation. The mixture was then centrifuged at 11292 ×g for 15 min at 4°C. The precipitated material was dialyzed with distilled water at 4°C for 72 h, and the protein fraction was lyophilized. This concentrated extract was used for the milk-clotting tests and zymogram assays.

### 2.9. Determination of Proteolytic Activity

Proteolytic activity was determined according to [14], using azocasein as the substrate and trichloroacetic acid as the precipitation agent. Formation of the chromophore was achieved by addition of 5 N KOH, and the color intensity was measured at 428 nm. One unit of protease activity (U) was defined as the amount of enzyme that produced a 0.01 absorbance difference between the reaction control and the sample, per minute of reaction, under the assay conditions. Protease activity was expressed as U/g of substrate.

### 2.10. Milk-Clotting Experiments

The clotting activities of the enzymatic extracts were determined according to [15]. The lyophilized extracts from *A. oryzae* CCBP 001, as well as the crude extract, were dissolved in 10 mM CaCl_2_ at concentrations of 20 mg/mL. The clotting time was measured after mixing 100 mL of each solution with 1 mL of reconstituted milk (12% (w/v) commercial skimmed milk powder dissolved in 10 mM CaCl_2_ at pH 6.5) and incubating at 37°C until clotting occurred.

### 2.11. Zymogram Analysis

The enzymatic activities of the *Aspergillus oryzae* CCBP 001 extracts were determined by zymography, adapted from [16]. A quantity of 8 mg of extract was added to 1 mL of 0.125 M Tris-HCl buffer (pH 6.8) containing 5% (w/v) SDS, 1% (w/v) sucrose, and 0.05% (w/v) bromophenol blue. A 10 *μ*L volume of each solution was loaded onto SDS-PAGE gel containing 0.1% (w/v) gelatin. Electrophoresis was performed with a 4.9% (w/v) polyacrylamide stacking gel in 0.125 M Tris-HCl buffer (pH 6.8) and a 15.4% (w/v) polyacrylamide resolving gel in 0.38 M Tris-HCl buffer (pH 8.8) containing 0.1% (w/v) SDS, at 4°C for 150 min at 500 V, 60 mA, and 30 W. After electrophoretic migration, the gel was washed twice with 2% (v/v) Triton X-100 for 30 min. The hydrolysis reaction then proceeded inside the gel during incubation at 37°C for 48 h in a bath of 0.05 M Tris-HCl buffer (pH 7.5) containing 15 mm CaCl_2_. The active enzymes were revealed as translucent bands after incubation of the gel, first in a mixture of 40% (v/v) ethanol, 10% (v/v) acetic acid, and 0.1% (w/v) R-250 Coomassie blue for 60 min and then in a destaining solution containing 30% (w/v) ethanol and 7.5% (v/v) acetic acid, with several washings.

## 3. Results and Discussion

### 3.1. Screening of Fungal Strains for Protease Production

**Figure 1:**
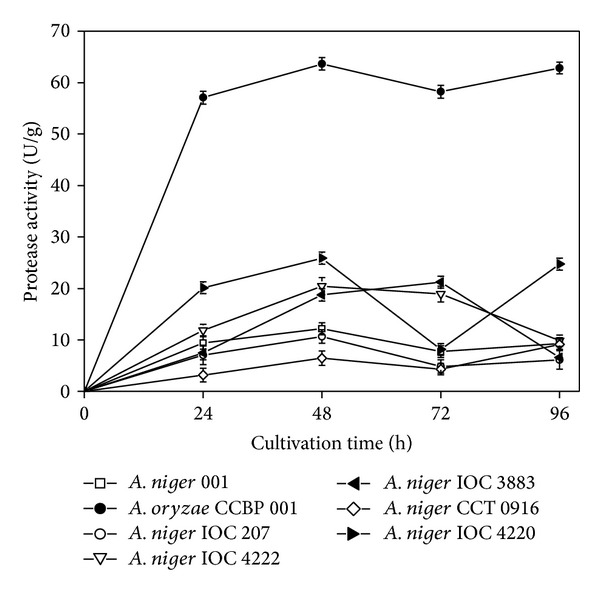
Protease production by different strains of *Aspergillus* cultivated under SSF for 96 h at 30°C using canola cake as substrate and an inoculum size of 10^6^ spores/g.

Protease production was evaluated for different *Aspergillus* strains cultivated under solid-state fermentation using canola cake as substrate, an inoculum size of 10^7^ spores/g, and incubation at 30°C for 96 h (Figure 1). All the strains tested were able to synthesize protease enzymes to some degree, and the temporal profiles were similar, with activity peaks after 48 h of cultivation. However, the *A. oryzae *CCBP 001 strain was clearly superior since it presented protease activity that was 3-fold higher than for the other strains after the first 24 h of cultivation, with maximum production of 64 U/g of substrate achieved after 48 h.

Sandhya et al. [7] screened fungi of the genera *Aspergillus *and *Penicillium* for neutral protease production under both SSF and SmF, using different agroindustrial wastes as substrates. The results were similar to those obtained in the present work, with a strain of *Aspergillus oryzae *(*A. oryzae* NRRL 1808) showing the best performance. According to Sumantha et al. [17], *A. oryzae* is the most important source of proteases. This fungus is considered to be nontoxigenic and has considerable potential as a new source of many industrially useful enzymes. It has a long history of extensive use in the food industry, which proves its safety [18]. Based on the results, the *Aspergillus oryzae* CCBP 001 strain was selected in studies to determine the effect of SSF process conditions on protease production using canola cake as solid substrate.

### 3.2. Effect of Substrate Initial Moisture Content

The water content of the substrate is one of the most important parameters affecting the efficiency of SSF processes. If the moisture level is too high, the void spaces in the solids are filled with water, resulting in oxygen limitation. At the other extreme, microorganism growth is restricted when the moisture content is too low [19]. The ideal moisture content for microbial growth is between 40 and 70%, depending on the microorganism and the substrate used for cultivation [20]. Identification of the optimal moisture content for a specific solid substrate is therefore crucial to ensure favorable growing conditions and maximize production of the metabolites of interest.

**Figure 2:**
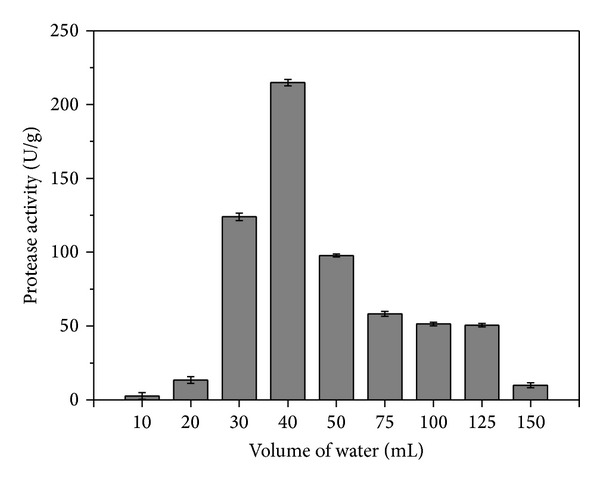
Effect of initial moisture content on protease production by *A. oryzae *CCBP 001 cultivated under SSF after 72 h at 30°C using canola cake as substrate and an inoculum size of 10^7^ spores/g.

The influence of initial moisture content on protease production during cultivation of *A. oryzae* CCBP 001 under SSF using canola cake as substrate was evaluated by adding different volumes of water to the solid medium (10, 20, 30, 40, 50, 75, 100, 125, and 150 mL to 100 g of canola cake). All the other process conditions were kept constant (inoculum size of 10^7^ spores/g and 96 h incubation at 30°C). Protease synthesis occurred under all the conditions tested. Initially, increasing the addition of water had little effect on protease production up to 40 mL, which optimized protease production (Figure 2). Further addition of water reduced protease synthesis while cultivations carried out using lower levels of water resulted in low enzymatic activities, possibly due to inadequate water availability (and consequently inefficient dissolution of nutrients from the substrate).

The effect of initial substrate moisture content on the production of proteases by other *Aspergillus* strains cultivated under SSF has been described previously. Battaglino et al. [21] used a combination of rice husk and rice bran as SSF substrate and assessed the effect of water addition on neutral protease production by *A. oryzae *NRRL 2160. The results were similar to those obtained here, with higher enzyme production for moisture contents of between 35 and 40%. Sandhya et al. [7] studied the effect of initial moisture content on protease production by *A. oryzae* NRRL 1808 cultivated under SSF using wheat bran as substrate and achieved a maximum enzyme yield of 30.5 U/g at 43.6% moisture content.

Based on these results, a ratio of 40 mL of water to 100 g of canola cake was selected for use during evaluation of the effects of the remaining SSF process parameters on protease production.

### 3.3. Effect of Inoculum Size on Protease Production

**Figure 3:**
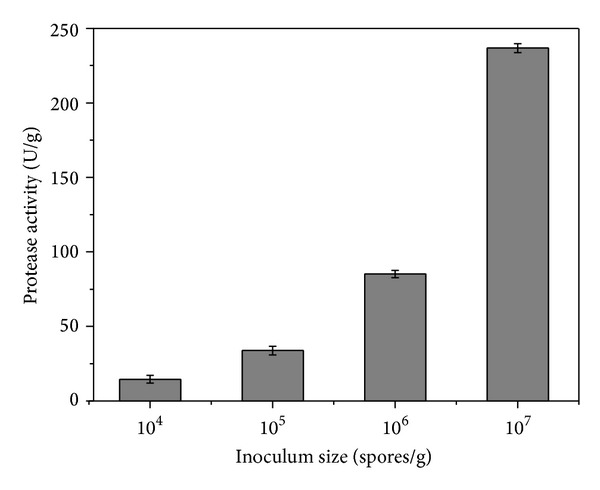
Effect of inoculum size on protease production by *A. oryzae *CCBP 001 cultivated under SSF after 72 h at 30°C using canola cake as substrate (moistened with 40 mL of water to 100 g of cake).

Inoculum size is a critical factor because it establishes the starting point for the fermentation process and influences the rates of both nutrient consumption and product formation. In these experiments, the inoculum size was varied between 10^4^ and 10^7^ spores/g of medium, maintaining all other process conditions constant (40 mL of water to 100 g of canola cake, 96 h of total fermentation at 30°C). Maximum protease activity was observed when the medium was inoculated with 10^7^ spores/g (Figure 3). Inoculum sizes of lower spores concentrations did not result in any significant protease activity. An inoculum size of  10^7^ spores/g was therefore used in the subsequent tests.

In earlier work, Sandhya et al. [7] studied the effect of inoculum concentration on protease production by *A. oryzae *NRRL 1808 grown on wheat bran and observed that concentrations higher than 8 × 10^8^ spores/g caused a reduction in production due to increased formation of biomass and rapid consumption of nutrients.

### 3.4. Effect of Temperature on Protease Production

**Figure 4:**
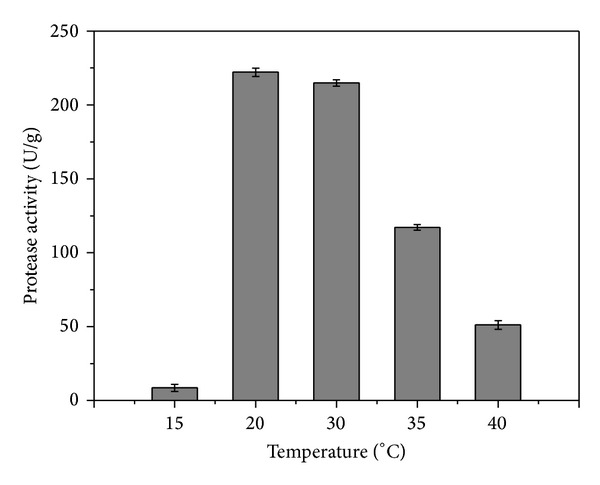
Effect of incubation temperature on protease production by *A. oryzae *CCBP 001 cultivated under SSF after 72 h using canola cake as substrate (moistened with 40 mL of water to 100 g of cake) and an inoculum size of 10^7^ spores/g.

Temperature is an important variable affecting microbial growth. Similar to the effects of moisture content and inoculum size, characterization of a particular microorganism in terms of the influence of temperature on product formation is essential in SSF bioprocess development. Cultivations were carried out at temperatures of 15, 20, 30, 35, and 40°C, using 10^7^ spores/g and addition of 40 mL of water to 100 g of cake (Figure 4). Protease synthesis was observed in all cases, although temperatures of 15 and 40°C resulted in poor fungal growth and enzyme production, reflecting the mesophilic nature of the microorganism. The highest protease activity after 72 h was observed for the medium incubated at 20°C. This temperature was therefore used during subsequent tests.

Thanapimmetha et al. [8] investigated the effect of temperature on protease production by *A. oryzae* cultivated under SSF using deoiled *Jatropha curcas* seed cake as substrate. The optimum temperature for the secretion of protease enzymes was 30°C, and there was a drastic decrease in protease production when the incubation temperature was raised to 35°C. Vishwanatha et al. [10] studied the production of protease by *A. oryzae* MTCC 5341 cultivated under SSF on wheat bran and found that the incubation temperature during growth had a significant influence on protease production. The optimum temperature for growth and protease production was 30°C, while a temperature of 25°C was suboptimal for growth, and growth ceased above 35°C.

### 3.5. Influence of pH on Protease Extraction and Activity

Besides optimizing the production process, it is also important to consider enzyme recovery during the extraction step. Identification of the most favorable conditions for enzyme extraction after cultivation using SSF involves consideration of several different variables, including solvent type, pH, solid to liquid ratio, temperature, type of agitation, and contact time, as well as the interrelations between these variables [22].

**Figure 5:**
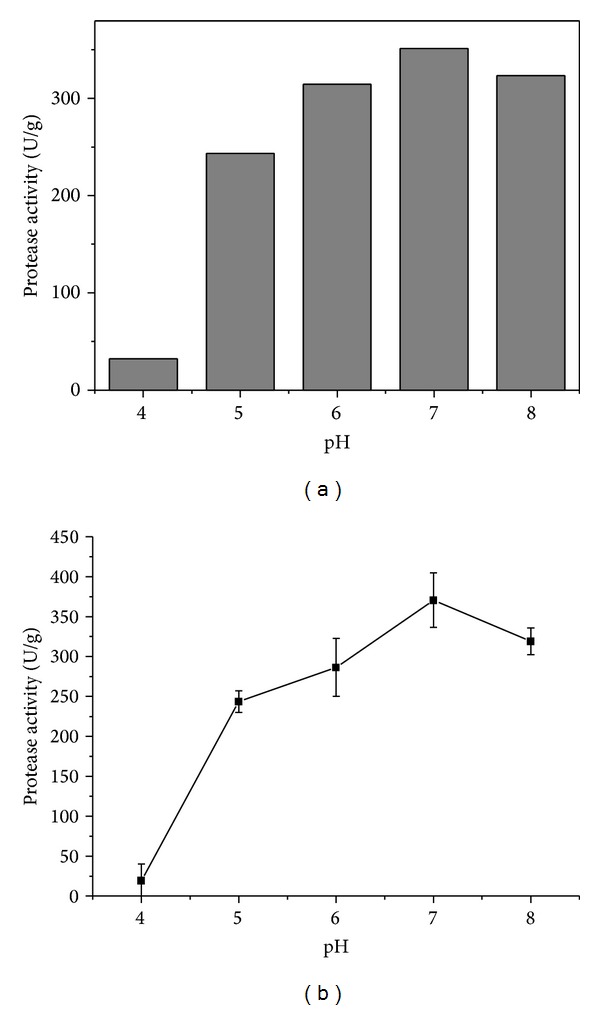
Effect of pH on the extraction (a) and activity (b) of protease.

Good recoveries of protease were obtained at all extraction buffer pH values, with the exception of pH 4.0 (Figure 5(a)). The highest recovery (354.3 U/g) was achieved using sodium phosphate buffer at pH 7.0. Ikasari and Mitchell [23] also found that the recovery of protease produced by *Rhizopus oligosporus* cultivated under SSF in rice bran increased as the pH of the extractant solution was increased, reaching a maximum at pH 7. Further pH increase to pH 9 reduced the recovery to only 16% of the maximum.

Enzymatic activity is strongly influenced by pH, since the active sites of enzymes are often composed of ionic groups whose conformation must be maintained in order to provide effective substrate binding and therefore enable catalysis to occur [24]. The substrate usually possesses one or more ionic groups that can bind preferentially to the enzyme. The influence of pH on protease activity was evaluated by performing activity assays in the pH range 4.0–8.0 (Figure 5(b)). Maximum proteolytic activity (371 U/g) occurred at pH 7.0, indicating that the enzyme produced was a neutral protease. According to Rao et al. [1], *A. oryzae* produces acid, neutral, and alkaline proteases. Fungal acid proteases have an optimal pH between 4 and 4.5 and are particularly useful in the cheese making industry. Fungal neutral proteases are most active at pH 7.0 and, given the accompanying peptidase activity and their specific ability to hydrolyze hydrophobic amino acid bonds, can supplement the action of plant, animal, and bacterial proteases in reducing the bitterness of food protein hydrolysates. Fungal alkaline proteases are also used in food protein modification.

The proteases produced by different microorganisms have characteristics that vary in terms of molecular size, optimal pH, and temperature (Table 1). It is therefore essential to characterize each particular enzyme in order to determine its properties and consequently its potential applications. According to Sumantha et al. [17], fungal neutral proteases, such as the one reported here, are the most important component of commercial fungal protease preparations, which have applications in the food, leather, animal feed, and pharmaceutical industries.

### 3.6. Protease Characterization in terms of Protein Coagulation Ability

Many extracellular proteases of microbial origin act similarly to chymosin in their ability to cleave caseinomacropeptide from k-casein, which triggers destabilization of the casein micelles. This mechanism induces milk clotting and can therefore be used in cheese production [25].

An ability to coagulate milk was shown by both the crude enzymatic extract obtained from the cultivation of *Aspergillus oryzae* CCBP 001 under the selected conditions and the concentrated form produced using the ammonium sulfate precipitation procedure. In the case of the crude extract, coagulation occurred after 150 min of incubation, indicating the action of the extract on milk proteins. The clotting time decreased from 25 to 5 min when the ammonium sulfate saturation condition was increased from 40% to 60 or 80%, confirming that the enzyme had been concentrated by the precipitation procedure. These results demonstrated that the protease extract from *A. oryzae* CCBP 001 has milk-clotting ability, although further studies concerning the quality of the enzyme and the product formed will be needed in order to confirm its usefulness in the dairy industry.

### 3.7. Zymogram Analysis of the Proteolytic Extract

**Figure 6:**
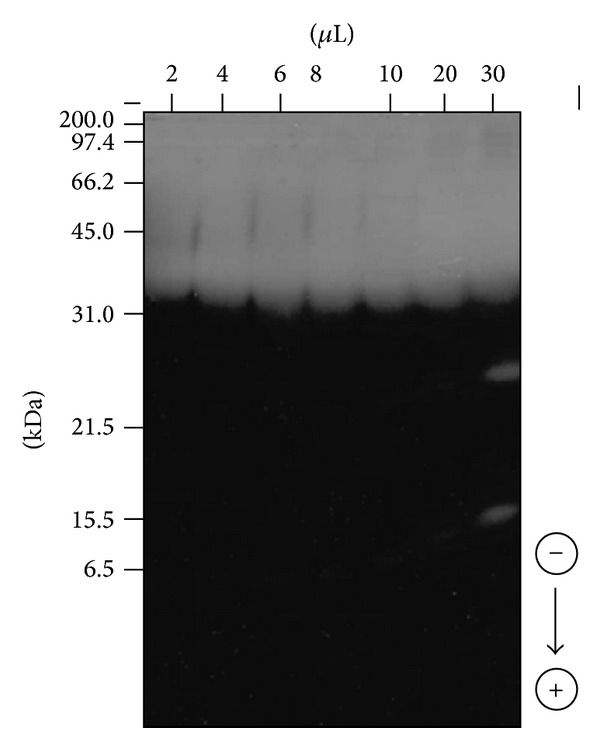
Zymogram analysis performed by sodium dodecyl sulfate polyacrylamide gel electrophoresis of extracts from *Aspergillus oryzae* CCBP 001. Experimental conditions: 15% (w/v) polyacrylamide, pH 8.8, and 0.1% (w/v) sodium dodecyl sulfate in the presence of gelatin. The molecular mass standards were myosin (200.0 kDa), *β*-galactosidase (116.2 kDa), phosphorylase b (97.4 kDa), bovine serum albumin (66.2 kDa), ovalbumin (45.0 kDa), carbonic anhydrase (31.0 kDa), trypsin inhibitor (21.5 kDa), lysozyme (15.5 kDa), and aprotinin (6.5 kDa).

Zymogram analysis of the concentrated enzymatic extract obtained under the selected conditions revealed several bands with proteolytic activity (Figure 6). Strong activity was observed in the region with molecular mass above 31 kDa, suggesting the presence of proteases. Two well-defined bands were also observed below 31 kDa when a higher concentration of the material was used. According to Vishwanatha et al. [26], *A. oryzae *possesses an extensive suite of hydrolytic genes, including 135 protease genes coding for alkaline, acid, and neutral proteases. This wide diversity of genes, added to the fact that variations in glycosylation can occur depending on the cultivation conditions, provides an explanation for the presence of the different proteases observed in the zymogram analysis.

Other *Aspergillus* species have been found to exhibit similar behavior, with a predominance of proteases having molecular weights greater than 31 kDa. Wang et al. [31] reported a 124 kDa protease from *A. fumigatus*, while Tremacoldi et al. [32] detected a 35 kDa alkaline protease produced by *A. clavatus*. Proteases smaller than 31 kDa have also been observed in *Aspergillus* extracts, such as the 23 kDa molecule obtained from *A. parasiticus* [30].

## 4. Conclusions

The results showed that canola cake is suitable for use as a substrate for protease production in SSF processes. Among the fungal strains tested, *A. oryzae* CCBP 001 performed best in terms of the synthesis of proteolytic enzymes. The cultivation conditions selected for protease production employed 40 mL of water to 100 g of canola cake, an incubation temperature of 20°C, and a spore concentration of 10^7^ spores/g. Maximum proteolytic activity (371 U/g) occurred at pH 7.0, which characterized the enzyme produced as a neutral protease. The concentrated protease extract was able to clot casein in 5 min. The enzymatic complex obtained from SSF of canola cake using *A. oryzae* CCBP 001 therefore has clear potential for applications in the food industry.

## Figures and Tables

**Table 1:** Properties of proteases produced by different microorganisms cultivated under SSF.

Microorganism	Substrate	Protease activity	MW (kDa)	pH	Temp. (°C)	Reference
*A. oryzae* MTCC 5341	Wheat bran	8.3 × 10^5^ U/g*	47	3-4	55	[26]
*Rhizopus oligosporus *	Rice bran	20.7 U/g	—	2	60	[23]
*A. fumigatus *	Fish flour	273 U/mL	88	7	60	[27]
*A. oryzae* NRRL 1808	Wheat bran	31.2 U/g	—	—	—	[7]
*Penicillium *sp.	Soybean cake	43 U/mL	—	6.5	45	[28]
*A. flavus *	Wheat bran	6.6 U/mL	—	7.5 and 9.5	—	[29]
*A. parasiticus *	Wheat bran	—	23	8	40	[30]
*A. oryzae* CCBP 001	Canola cake	371 U/g	31–200	7	—	This work

*A different activity assay was used.
